# Relationship between Tibial conformation, cage size and advancement achieved in TTA procedure

**DOI:** 10.1186/s12917-018-1433-0

**Published:** 2018-03-20

**Authors:** R. L. Meeson, L. Corah, M. C. Conroy, I. Calvo

**Affiliations:** 1 Department of Clinical Sciences and Services, Royal Veterinary College, Queen Mother Hospital for Animals, Hawkshead, Lane, North Mymms, Hertfordshire, UK; 2 Veterinary Epidemiology, Economics and Public Health (VEEPH) Group, Royal Veterinary College, Hawkshead, Lane, North Mymms, Hertfordshire, UK

## Abstract

**Background:**

Previous studies have suggested that there is a theoretical discrepancy between the cage size and the resultant tibial tuberosity advancement, with the cage size consistently providing less tibial tuberosity advancement than predicted. The purpose of this study was to test and quantify this in clinical cases. The hypothesis was that the advancement of the tibial tuberosity as measured by the widening of the proximal tibia at the tibial tuberosity level after a standard TTA, will be less than the cage sized used, with no particular cage size providing a relative smaller or higher under-advancement, and that the conformation of the proximal tibia will have an influence on the amount of advancement achieved.

**Results:**

One hundred sixty-four dogs met the inclusion criteria. The mean percentage under-advancement was 15.5%. All dogs had an advancement less than the stated cage size inserted. An association between the proximal tibial tuberosity angle (increased in cases with low patellar tendon insertion), and percentage under-advancement was found, with an increase of 0.45% under-advancement for every 1 degree increase in angle a (*p* = 0.003). There was also evidence of a difference between the mean percentage under-advancement in breeds (*p* = 0.001) with the Labrador having the biggest under-advancement. Cage size (*p* = 0.83) and preoperative tibial plateau angle (*p* = 0.27) did not affect under-advancement.

**Conclusions:**

The conformation of the tibial tuberosity and therefore the relative cage positioning have an impact on mean percentage under-advancement of the tibial tuberosity after standard TTA. In all evaluated cases, the advancement of the tibial tuberosity was less than intended by the cage size selected.

## Background

Cranial cruciate ligament disease (CCLD) is a significant cause of morbidity in dogs, leading to lameness, muscle atrophy and osteoarthritis [1, 2]. The underlying aetiology of CCLD is poorly understood and a myriad of treatment options have been proposed. Over the last two decades, attention has focused on providing dynamic stifle stability by tibial osteotomy [3–8]. One of the newer surgical techniques, the tibial tuberosity advancement (TTA), has been shown to reduce tibial translation in the CCL-deficient stifle and to provide good clinical outcomes [9]. However, issues including residual femoro-tibial instability and high rate of late meniscal injuries have been raised [10]. Proposed explanations include flaws in the biomechanical principles and technical errors in planning or execution [10].

Currently, the translation distance required of the tibial tuberosity to advance the patellar tendon angle to a 90 degree tangent to the tibial plateau is measured pre-operatively by one of several methods, at the level of the patellar tendon insertion point [11]. A cage size matching the required translation distance is inserted into the osteotomy with the intention of advancing the patellar tendon insertion the intended distance (cage size) and subsequently altering the patella-tendon angle (PTA) to the desired advancement.

However, it is has been shown that there is a theoretical discrepancy between the cage size and the resultant tibial tuberosity advancement, with the cage size consistently providing less tibial tuberosity advancement than predicted [12]. This occurs as the advancement distance required is measured in a direction parallel to the tibial plateau, whereas the plane of the tibial crest osteotomy is not perpendicular to the tibial plateau. The result is that the cage size deemed appropriate will give a smaller advancement than expected, as the cage is positioned within the osteotomy [12]. This theoretical discrepancy has been demonstrated with one of the many TTA variations, the modified Maquet, which provided 30% less advancement than intended [13]. It is important to mention that the modified Maquet procedure, as well as the theoretical model, do not allow the tibial tuberosity to migrate proximally, as we see with the standard TTA procedure. Furthermore, significant breed variation in proximal tibial conformation is a well-known phenomenon, with some breeds having a relative lower or higher patellar tendon insertion [14]. As TTA cages are trapezoid in shape, and narrow from proximal to distal, the effective advancement as measured at the tibial tuberosity will be more or less than the selected cage width depending on the relative position of the widest aspect of the cage and the tibial tuberosity. A small case series has also been reported, whereby the cage was intentionally positioned very distal to produce a greater advancement [14].

The hypothesis was that the actual tibial tuberosity advancement, as measured by the widening of the proximal tibial at the level of the insertion of the patellar tendon, will be less than the advancement size deemed by the cage size selected. The second hypothesis was that advancement will be influenced by the conformation of the proximal tibial. The aim was to measure the radiographic advancement of the insertion of the patellar tendon and compare to the cage size selected in patients that had undergone a standard TTA procedure. The second aim was to measure the conformation of the proximal tibial and evaluate whether there is a relationship to the under advancement seen.

## Methods

Medical records of dogs that underwent a standard TTA at the Royal Veterinary College from September 2010 to May 2014 were reviewed to identify dogs treated for cranial cruciate ligament disease with a TTA procedure. Data included breed, age, gender, and cage size. Additional inclusion criteria were the availability of complete medical records, and pre-op and post-op radiographs. Dogs were excluded if the radiographic positioning was poor, caudal margin of the medial and lateral tibial condyles were separated by more than 4 mm or if pre-operative radiographs were not positioned at 135 ± 10° degrees of flexion. At least the proximal third of the tibia and a radiodense 10 cm radiographic marker must have been present. Non-standard TTA surgeries (additional implants), were omitted.

**Fig. 1:**
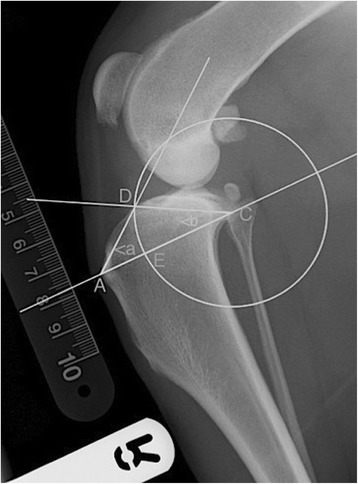
Standard straight mediolateral stifle radiograph illustrating the landmarks for tibial conformation assessment. A: most proximal point of the margo-cranialis tibiae; C: most caudal point of the tibial plateau, represented by the midpoint between the medial and lateral tibial condyles; D: most cranial point of the tibial plateau; E: cross point of a circle with the centre C and the radius CD, and the line AC. Proximal tibial tuberosity angle (PTTA): angle <a formed by DAE. Tibial plateau angle (TPA): angle <b, formed by ACD (15)

**Fig. 2:**
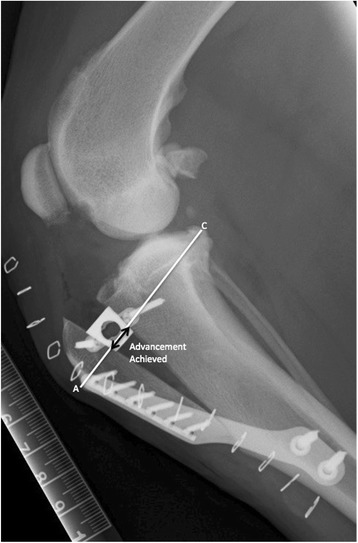
Standard mediolateral post-TTA stifle radiograph. The achieved advancement of the patellar tendon insertion was measured along the post-operative line AC (A = most proximal point of margo cranialis tibiae; C = most caudal point of the tibial plateau, represented by the midpoint between the medial and lateral tibial condyles post TTA). The advancement along that line was measure from the cranial to the caudal aspect of the osteotomy created

All cases had a standard TTA procedure performed using titanium TTA implants (Kyon, Zurich, Switzerland) [9]. Straight medio-lateral pre-operative and post-operative radiographs were digitally evaluated (Osirix®, Geneva, Switzerland), three times by two evaluators. The evaluators were blinded to the outcome. Proximal tibial conformation measurements were made on the pre-operative radiographs, using calibrated measures and previously described landmarks [15]. In brief, point A was the most proximal point of the margo cranialis tibiae (insertion of the patellar tendon); point C was the most caudal point of the tibial plateau, represented by the midpoint between the medial and lateral tibial condyles; point D was the most cranial point of the tibial plateau; point E: cross point of a circle with the center C and the radius CD, and the line AC. The proximal tibial tuberosity angle (PTTA): angle a, was formed by DAE. Tibial plateau angle (TPA): angle b, was formed by ACD (Fig. 1). Preliminary planning showed that many post-operative radiographs were taken at angles other than 135 degrees, and it is noted that true achievement of 135 degrees stifle flexion is variable dependent upon method [16]. The effect of tibial tuberosity conformation was therefore used to assess the advancement of the tibial tuberosity by measuring the width of the tibial osteotomy created. This measure therefore would not be affected by the degree of stifle flexion when the radiograph was taken. Using the immediate post-operative radiographs (Fig. 2), the advancement was calculated by repeating line A-C on the post-operative radiograph, and then measuring the osteotomy width along that line A-C. The achieved advancement was expressed in absolute numbers and as a percentage of the desired advancement (cage size placed). All measurements were repeated in triplicate and means were used for analysis.

Data was collected and cleaned in a spreadsheet (Excel 2013, Microsoft Corp.), and exported to Stata 13.1. (StatCorpLP). Continuous data was checked for normality; non-normal data was transformed to ensure normality. Dog breeds were grouped as cross breeds, Rottweilers, Golden Retrievers, Labradors and ‘other purebreds’, and cage sizes were assessed individually and grouped as small-medium (sizes 6 and 9), and large-giant (sizes 12 and 15). Explanatory variables were described. Student’s t-test, one-way analysis of variance and linear regression were used to assess the crude association with under-advancement (percentage and absolute). Standard methods were used to calculate 95% confidence intervals [17]. Linear regression was used to evaluate risk factors for under-advancement. In all models, variables that were loosely associated (*P* < 0.2) were taken forward to a multivariable model. Backward stepwise elimination was used to build the model. Significance was set at the 5% level. All variables dropped from the model were assessed for confounding and biologically plausible interaction. For linear models, homoscedasticity, normality of residuals and linearity were confirmed using described methods [18]. Outliers were checked for and influential observations were evaluated using Cook’s distance. Collinearity between explanatory variables was evaluated by examining the Variance Inflation Factor. For logistic models, model fit was assessed using the Hosmer-Lemeshow test and evaluation of the ROC curve [19]. Covariant patterns were assessed for leverage using the delta beta and delta deviance statistics [18]. Cage size was integrated into the model as it was of a-priori interest. The linear regression model, provides a y = mx + c plot, whereby ‘y’ is the under advancement, ‘c’ is the model constant, and ‘m’ refers to the regression coefficient of each variable, and ‘x’ the variable measured. This relationship was used as a predictive model that could account for varying patella insertion heights to improve pre-operative planning, allowing a correction of the cage size initially selected based on the patellar insertion point.

## Results

One hundred sixty-four dogs met the inclusion criteria, aged 15-138 months (mean 63.7). The breed distributions were cross breeds (21.3%), Retrievers (17.7%), Rottweilers (11.0%), and other pure breeds (50%). Dogs were male in 55% and female in 45%. The left cruciate was affected in 49% and the right in 51%. Size 6 mm cages were placed in 7%, size 9 mm in 46%, size 12 mm in 40% and size 15 mm in 7% of dogs.

The mean percentage under-advancement was 15.5% (95% CI 14.3 – 16.6). All dogs had an advancement of the tibial tuberosity that was less than the intended cage size, and therefore all dogs would be under-advanced. Based on the univariate statistical screening of continuous exposure variables (Table 1), angle b (TPA) did not influence the under-advancement in % (*p* = 0.27) or absolute terms (*p* = 0.624). However an association between angle a and percentage under-advancement was found, with an increase of 0.45% under-advancement for every 1 degree increase in angle a (*p* = 0.003). An association was also present between angle a and absolute under-advancement with an increase of 0.05 mm of under-advancement for every 1 degree increase in angle a (*p* = 0.01). Age and angle b were not associated with the occurrence of under-advancement at significance level of *p* < 0.2 and thus were not included in the multivariable modelling process. Univariate statistical screening of categorical exposure variables (Table 2) showed that although individual cage size was not associated with percentage or absolute (mm) under-advancement, dogs with cage sizes 6 and 9 mm (mean under-advancement 1.34 mm), had a significantly absolute smaller under-advancement than dogs with cage sizes 12 and 15 mm (mean of 1.93 mm) (*p* < 0.001). There was also evidence of a difference between the mean percentage and absolute under-advancement between breeds (*p* = 0.001 and *p* = 0.007). Sex was also associated with the occurrence of under-advancement at significance level of *p* < 0.2 and thus were included in the multivariable modelling process.

**Table 1:** Descriptive statistics for continuous exposure variables and univariable analysis for % under advancement and mm under advancement

				Univariable Analysis for % under advancement				Univariable analysis for mm under advancement			
Variable	Mean	Standard deviation	95% Confidence Interval	Regression coefficient	95% Confidence interval	*p*-value	adjusted R^2^	Regression coefficient	95% Confidence interval	*p*-value	adjusted R^2^
Angle a	33.45	3.84	32.86 - 34.05	0.45	0.16 - 0.74	0.003	0.05	0.05	0.02 - 0.09	0.001	0.06
Constant				0.38	- 9.43 - 10.19	0.94		−0.2	- 1.31 - 0.91	0.7	
Angle b	30.25	4.35	29.58 - 30.92	0.15	−0.12 - 0.41	0.27	0.001	0.007	- 0.02 - 0.04	0.62	−0.005
Constant				11.05	2.9 - 19.11	0.007		1.38	0.47 - 2.30	0.003	
Age	4.89	2.24	4.55 - 5.24	0.2	- 0.32 - 0.71	0.46	−0.003	−0.01	- 0.07 - 0.05	0.68	−0.005
Constant				14.53	11.77 - 17.29	< 0.001		1.67	1.36 - 1.98	< 0.001

**Table 2:** Categorical exposure variables. Descriptive data and univariate analysis with % under-advancement and mm under-advancement as outcomes

Variable		*n*(%) (*N* = 164)	Mean under advancement (%)	Standard deviation	95% Confidence Interval	*p*-value	Mean under advancement (mm)	Standard deviation	95% Confidence Interval	*p*-value
Cage Size	6 mm	12 (7.3%)	15.36	7.58	13.76-16.97	0.83	1.34	0.7	CI 1.19-1.48	< 0.001
	9 mm	76 (46.3%)								
	12 mm	65 (39.6%)	15.61	7.27	13.95-17.27		1.93	0.88	CI 1.72 - 2.13	
	15 mm	11 (6.7%)								
Sex	Male	90 (54.9%)	16.29	7.33	14.75 - 17.82	0.12	1.73	0.84	1.55 - 1.90	0.05
	Female	74 (45.1%)	14.5	7.45	12.77 - 16.22		1.47	0.83	1.27 - 1.66	
Neutered	Entire	29 (17.7%)	14.96	14.96	12.76 - 17.15	0.68	1.67	0.76	1.38 - 1.96	0.68
	Neutered	135 (82.3%)	15.59	15.59	14.28 -16.91		1.6	0.86	1.45 - 1.74	
Breed	Crossbreed	35 (21.3%)	14.07	7.44	11.51 - 16.62	0.001	1.53	0.87	1.35 - 1.73	0.007
	Other Purebred	82 (50%)	14.59	7.62	12.92 - 16.27		1.41	0.83	1.13- 1.70	
	Rottwieller	18 (11.0%)	15.91	5.66	13.09 - 18.72		1.76	0.77	1.38 - 2.15	
	Golder Retriever	15 (9.2%)	18.71	5.86	15.47 - 21.96		1.99	0.75	1.58 - 2.41	
	Labrador	14 (8.5%)	20.19	7.62	15.79 - 24.58		1.91	0.74	1.48 - 2.33	

On Multivariate statistical analysis, breed remained significantly associated with percentage under-advancement, with an increase of 1.61% mean under-advancement or 0.17 mm absolute under-advancement as breed changed from crossbreed – other purebred – Rottweiler – Golden Retriever – Labrador (*p* = 0.001). Similarly, angle a remained significantly associated with under-advancement, with an increase in absolute under-advancement of 0.05 mm for each 1 degree increase in angle a (*p* = 0.001), and an increase in percentage under-advancement of 0.45% under-advancement for every 1 degree increase in angle a (*p* = 0.002). Cage size (6 and 9 mm vs 12 and 15 mm) remained significantly associated with absolute under-advancement (*p* < 0.001) (Table 3).

**Table 3:** Multivariable Linear Regression for under-advancement

	Variable	Regression Coefficient	95% Confidence Interval	*p*-value
Model A: Precentage underadvancement as outcome	Angle a	0.45	0.17 - 0.73	0.002
	Breed	1.61	0.68 - 2.54	0.001
	Constant	−1.98	- 11.56 - 7.60	0.69
	Adjusted R^2^			0.1
Model B: absoulte underadvancement as outcome	Cage size	0.58	0.36 - 0.81	< 0.001
	Angle α	0.05	0.02 - 0.08	0.001
	Breed	0.17	0.08 - 0.27	0.001
	Sex	−0.22	- 0.45 - 0.01	0.06
	Constant	−0.48	- 1.50 - 0.54	0.36
	Adjusted R^2^			0.23

From the data on the tibial conformation, a predictive model (adjusted R^2^ = 0.05), can be used based on pre-surgical measurements to correct for the size of cage: additional % advancement required = 0.38 + (0.45 x dog’s angle a).

## Discussion

This study supports the hypothesis that the conformation of the tibial tuberosity has an influence on the advancement of the tibial tuberosity in TTA surgery. It also supports the suggestion that due to the trapezoid shape of the TTA cage, and the surgical cage position relative to the tibial tuberosity, that dogs were under-advanced when compared to the cage size used. The average under-advancement by this measure was 15%, which was half of that reported by Kapler et al. (2015). Further differences could be accounted for by the technical differences between a ‘standard’ Kyon TTA and a modified Maquet procedure where the distal aspect of the osteotomy remains attached to the rest of the tibia and proximal displacement of the osteotomised tibial tuberosity is prevented. Etchepareborde [12] showed in a theoretical model that the cage size selected from pre-operative templating does not provide the expected advancement of the PTA. This was due to the advancement from the cage being orientated in a different direction to the pre-operative measurement. They showed this would result in an under-advancement such that the PTA would not be 90 degrees post-operatively, and therefore on going stifle instability could result. Their study being theoretical assumed a standardised tibial tuberosity shape with a consistent position of the tibial tuberosity. Additionally, they did not account for the rise of the tibial tuberosity seen with complete osteotomies. Our study has further shown that the intended cage size results in less advancement than expected due to the conformation of the tibial tuberosity. Therefore, there is an addition source of potential under-advancement error. PTA measurements were not performed post-operatively in this study as the degree of stifle flexion was quite variable and it is currently unknown if this will change the measure PTA. Based on Etchepareborde’s work however [12], it is likely that a smaller than expected advancement of the osteotomy will further reduce the post-operative PTA. This however, does not remove the significant finding that the osteotomy translation distance is less than expected by the cage size inserted and therefore leads to a further cause of under-advancement.

As predicted, tibial conformation as measured by angle a had a significant effect on the degree of under-advancement. As the insertion of the patellar tendon became more distal, (increasing angle a), the amount of under-advancement increased. This phenomenon is a result of the aforementioned trapezoid conformation of the TTA cages. For example, when a 15 mm advancement is calculated in pre-operative planning, a 15 mm cage will be normally selected. This cage is 15 mm wide at its most proximal aspect, and less distally. The position of the proximal aspect of the cage relative to the tibial tuberosity is therefore critically important. The current Kyon recommendation is to place the cage around 2 mm distal to the tibial plateau of the parent tibia. Clearly the conformation of the tibial tuberosity and the degree of proximal displacement of the tuberosity will affect how the tibial tuberosity aligns to the proximal cage. Overall every 1 degree increase in angle alpha leads to an increase in under-advancement by 0.45%. From the data analysed, a predictive model could account for some of the effect of varying patellar insertion heights. As an initial step in pre-operative planning, this may have some use in alerting the surgeon to cage sizes that may be more appropriate; (Additional % advancement required = 0.38 + (0.45 x dog’s angle a)), to achieve the intended advancement at the patellar tendon insertion point, and thus the correct advancement. However, although this is a statistically significant relationship, this model did not entirely account for the under-advancement seen, but may be helpful for ameliorating it.

In order to further investigate the relevance of proximal tibial conformation in tibial tuberosity advancement we decided to evaluate the effect of the tibial plateau angle. Inauen in 2009 described the use of angle b as an alternative method to measure the tibial plateau angle. In our study, angle b was not associated with under-advancement. However, angle b depends considerably upon the relative position of the tibial tuberosity and may therefore carry the risk of being influenced by a dependent value. This index of TPA may not be directly comparable to traditional methods and therefore conclusions based on angle b should be viewed with caution.

Furthermore and since the tibial tuberosity is one of the landmarks used to calculate angle b, the fact that we did not find a relation between angle b and under-advancement is somehow an unexpected finding. It is possible that there is a morphological relationship between the insertion of the patellar tendon (angle a) and the TPA (angle b) in the proximal tibia, which could confound the expected relationship between them. It therefore could be that high insertions are differentially associated with a particular TPA conformation, and hence as our measurements were made from the clinical cases and were not theoretical models, it may explain why we did not see significant changes in angle b with under-advancement.

Etchepareborde, showed a relationship of under-advancement to the TPA, which was not found in this study [12]. This difference may be explained by the issues described in the previous paragraph or by the fact their study assumed the osteotomy was absolutely parallel to the tibial axis, that there was no proximal migration of the osteotomised tibial tuberosity and that the proximal aspect of the cage was always placed at the level of the tibial tuberosity.

Breed was a risk factor for percentage under-advancement, with the lowest risk in crossbreeds and the highest in Labradors. Breed may influence tibial conformation, however breed remained significant independent of tibial crest conformation (angle a) indicating another effect that we cannot explain. A further possibility could be the role of surgeon on cage positioning, which may have been in turn affected by the breed of dog they are operating on, as this was not blinded, however this is pure conjecture.

The fact that we found that grouped cage size (6 and 9 mm vs 12 and 15 mm) were significantly associated with absolute under-advancement has no clinical relevance. The absolute advancement provided by small cages will be less than the absolute advancement provided by big cages. The meaningful information in relation to this variable (cage size) is that the % under-advancement was not affected by individual or grouped cage sizes.

The clinical significance of the findings of this study are unclear, however if we follow the current cage selection recommendations, it appears that all dogs will be under-advanced. The TPLO procedure aims to achieve a post-operative tibial plateau angle of five to 6.5 degrees, however good clinical outcome in dogs have been reported up to 14 degrees [20]. Potentially, a degree of TTA under-advancement may be clinically tolerable [21]. A larger study with clinical outcome evaluation is required to resolve this question.

This paper has several limitations and should be interpreted carefully. Firstly, radiographic measurements were derived from pre- and post-op radiographs. Positioning and rotation can affect magnification and cause radiographic lengthening or shortening, altering the measurements made. To improve precision, measurements were performed in triplicate using the straightest views, with calibrated measurements from a calibration object. It is also known that there can be variation in measurements of identification of anatomical landmarks for tibial plateau angle measurements [22–25]. Secondly, post-operative PTA was also not measured, as post-operative radiographs were not all positioned at 135 ± 10 degrees. It therefore remains to be proven if conformation of the tibial tuberosity results in a change of post-operative expected PTA. Irrespective, we know from other studies that reducing the osteotomy advancement does reduce the PTA [12]. Thirdly, the kerf of the blade was not taken into consideration as a factor influencing the under-advancement, however the same blade thickness was used in all cases, minimising variability between cases. And lastly, the degree of proximal translation of the tibial tuberosity was not assessed in this paper. Recent literature has shown that failure to translate the tibial tuberosity proximally during TTA results in significant under-advancement as compared to shifting the tibial tuberosity proximally by 6 mm [26]. However in all our cases we allowed the tibial tuberosity to ‘jump-up’ without forcing it, reproducing the current clinical recommendation on how to perform TTAs and minimising the possibility of having 6 mm differences in proximal tibial tuberosity translations between cases.

## Conclusion

Despite the limitations, this study has made some significant observations. Firstly, current cage design and surgical techniques consistently result in under-advancement as measured by desired translation (cage size), of the tibial tuberosity at the level of the patellar tendon insertion. The conformation of the tibial tuberosity influences the degree of under-advancement and should be taken into consideration during planning. A corrective equation has been suggested and may help improve cage size selection after templating distances have been calculated, and special consideration should be given to the higher risk Labradors.

## Abbreviations

CCLD: Cranial cruciate ligament disease
PTA: Patella-tendon angle
PTTA: Proximal tibial tuberosity angle
TPA: Tibial plateau angle
TTA: Tibial tuberosity advancement
